# Lipid peroxidation and total antioxidant capacity in vitreous, aqueous humor, and blood samples from patients with diabetic retinopathy

**Published:** 2011-05-07

**Authors:** Raffaele Mancino, Donato Di Pierro, Chiara Varesi, Angelica Cerulli, Alessandra Feraco, Claudio Cedrone, Maria Dolores Pinazo-Duran, Massimiliano Coletta, Carlo Nucci

**Affiliations:** 1 Ophthalmology Unit, Department of Biopathology, Tor Vergata University of Rome, Rome, Italy; 2 Department of Experimental Medicine and Biochemical Sciences, Tor Vergata University of Rome, Rome, Italy; 3 Department of Pharmacobiology, University of Calabria, Cosenza, Italy; 4 Ophthalmic Research Unit “Santiago Grisolia”University Hospital Dr Peset, Valencia, Spain

## Abstract

**Purpose:**

To evaluate levels of malondialdehyde and the total antioxidant capacity (TAC) in the blood, aqueous humor, and vitreous bodies of diabetic and nondiabetic patients. We also measured the blood energy charge potential (ECP).

**Methods:**

We examined 19 patients with type 2 diabetes mellitus and diabetic retinopathy. Ten were scheduled for cataract surgery and pars plana vitrectomy because of proliferative diabetic retinopathy (PDR). The other nine, with mild nonproliferative PDR (NPDR), and fourteen nondiabetic, age-matched subjects enrolled as a control group were scheduled for cataract surgery and vitrectomy because of epiretinal membranes. Blood, aqueous humor and vitreous body samples were collected at the time of surgery. Malondialdehyde concentrations and blood ECP were measured with high-performance liquid chromatography. The TAC of the samples was estimated with the oxygen radical absorbance capacity method.

**Results:**

The level of blood and vitreous malondialdehyde in the PDR group was significantly higher compared to controls and to NPDR patients. PDR patients also had lower levels of TAC at the vitreous body and aqueous humor level, but not at the blood level, compared to controls and with NPDR patients. In all diabetic patients, the blood ECP values were significantly lower, compared to control subjects.

**Conclusions:**

Our data support the hypothesis that oxidative stress and the decrease of antioxidant defenses are associated with the progression of diabetic retinopathy to its proliferative form. Antioxidant supply may have the effect of correcting oxidative stress and inhibiting disease progression.

## Introduction

In vitro and in vivo studies suggest that oxidative stress is increased in diabetes and that this plays an important role in the pathogenesis of diabetic complications, including retinopathy. Increased superoxide levels have been demonstrated in the retinas of diabetic rats and in retinal cells incubated in high-glucose media [1,2]. The retinas of rats with experimentally induced diabetes also display more extensive membrane lipid peroxidation and oxidative DNA damage, which are the consequences of reactive oxygen species (ROS)-induced injury [1,3-5]. Interestingly, oxidative stress contributes not only to the onset of diabetic retinopathy, but also to its persistence after glycemic control has been reestablished. This phenomenon, which is referred to as “metabolic memory” [6], has been attributed to the accumulation of damaged molecules and ROS that are not easily eliminated by the restoration of normoglycemia.

Oxidative stress reflects excess formation and/or impaired removal of ROS. Therefore, adequate levels of the antioxidant enzymes responsible for scavenging free radicals are essential for redox homeostasis. Retinal levels of enzymes such as superoxide dismutase (SOD), glutathione reductase, glutathione peroxidase, and catalase are decreased in animals with experimentally induced diabetes [3,7]. As a consequence, intraretinal levels of the potent antioxidant, glutathione, are also decreased in diabetic rats [8]. In humans, Pan et al. [9] measured oxidative stress parameters in the blood, and their findings strengthen the hypothesis that an increased presence of oxidative species contributes to the onset and advancement of diabetic retinopathy. However, apart from this interesting study, very little research has been done on the oxidative and antioxidant capacities of body fluids in patients with diabetes or on how these capacities correlate with the stage of the disease.

To address this issue, we analyzed samples of blood and aqueous and vitreous humors from diabetic patients with proliferative and nonproliferative forms of retinopathy and nondiabetic controls. Each specimen was subjected to high performance liquid chromatography (HPLC) to determine levels of malondialdehyde (MDA), which is produced during phospholipid peroxidation [10] and the activation of the arachidonate cycle [11]. MDA is widely regarded as a marker of peroxidative damage to cell membranes induced by physical or chemical oxidative stress [12]. The total antioxidant capacity (TAC) of each sample was also determined with the oxygen radical absorbance capacity (ORAC) method and according to energy charge potential (ECP), a biomarker of tissue energy status that was assessed in blood samples.

## Methods

The study was approved by the institutional review board of the University Hospital Tor Vergata. The study followed the tenets of the Declaration of Helsinki. All participants provided informed consent after receiving an explanation of the nature and possible consequences of the study. The study group consisted of 19 patients with type 2 diabetes mellitus (nine males and ten females; mean age: 72±1.7 years) recruited from the Ophthalmology Unit of the Tor Vergata University of Rome Medical Center. All diagnoses of type 2 diabetes were based on criteria recommended by the World Health Organization, and all patients were being treated with dietary measures and insulin. Retinopathy was classified by an independent ophthalmologist using fundus photography and fluorangiographic imaging. On the basis of his findings, patients were divided into two groups. Group 1 included ten patients with mild nonproliferative retinopathy (NPDR), and group 2 consisted of nine patients with proliferative retinopathy (PDR). The mean duration of diabetes for NPDR and PDR patients was 16.9±4.2 years and 21.3±6.1 years respectively. For comparison purposes, we also enrolled a control group consisting of 14 age-matched nondiabetic subjects (6 males and 8 females, mean age 70±2.1 years).

Group 1 patients and control subjects were scheduled for cataract surgery and vitrectomy to eliminate epiretinal membranes, with or without macular holes. Group 2 patients were scheduled for cataract surgery and pars plana vitrectomy for long-standing (>3 months) or recurrent vitreous or preretinal hemorrhage, or for tractional retinal detachment involving or threatening the macula. In this group, laser photocoagulation was performed at an earlier stage of the disease (but not in the preceding three months) in all patients.

Study candidates were excluded for any of the following reasons: smoking, glaucoma, liver disease, severe nephropathy, cancer, collagen diseases, acute or chronic infections, fever, congestive heart failure, or use of oral antioxidant supplements.

### Sample collection

Samples of blood, aqueous humor, and vitreous were collected from all participants on the day of surgery. Venous blood samples were drawn after an overnight fast. The samples were placed on ice and centrifuged within 1 h (2,800× g at 4 °C for 15 min), and the supernatants were stored at −20 °C until analyzed. Aqueous humor samples (0.1–0.2 ml) were rapidly collected at the beginning of cataract surgery with a 27-gauge needle on a tuberculin syringe. Special care was taken to avoid blood contamination. Samples were immediately cooled and stored at −70 °C. Vitreous humor (300–400 μl) was collected from each patient before infusion started. Samples were placed in individual cryo-Eppendorf tubes, stored at −70 °C, and assayed within 2 weeks of collection.

### Quantitative analysis of malondialdehyde and energy state

Blood, aqueous humor, and vitreous samples were collected in sterile tubes. Ice-cold 1.2 M HClO_4_ (1:2, w/w) was added to the blood samples to deproteinize erythrocytes. All samples were centrifuged at 20,690× g for 10 min at 4 °C, neutralized by adding 5 M K_2_CO_3_ in ice, filtered through a 0.45 μM Millipore-HV filter (Millipore Corporation, Billerica, MA), and subjected to HPLC. The ion-pairing method was used for simultaneous direct determination of MDA and adenine nucleotide levels in 100 μl of a perchloric acid extract from each sample [12]. We used a Vydac 250×4.6 mm, 5-μm particle size column with its own guard column (Eka Chemicals AB, Bohus, Sweden) and tetrabutylammonium hydroxide as the ion-pairing reagent. Briefly, metabolites were separated by creating a step gradient (adapted to the column size [12] with two buffers: buffer A was 10 mM tetrabutylammonium hydroxide, 10 mM KH_2_PO_4_, 0.25% methanol, pH 7.00; buffer B was 2.8 mM tetrabutylammonium hydroxide, 100 mM KH_2_PO_4_, 30% methanol, pH 5.50). The gradient was 10 min, 100% buffer A; 3 min, 90% buffer A; 10 min, 70% buffer A; 12 min, 55% buffer A; 15 min, 45% buffer A; 10 min, 25% buffer A; and 5 min, 0% buffer A. A flow rate of 1.2 ml/min was maintained throughout each run, and the column temperature was maintained at 23 °C with the aid of water-jacketed glassware. The HPLC apparatus consisted of a Surveyor LC Pump (ThermoFinnigan Italia, Rodano, Milan, Italy) connected to a Surveyor PDA Detector (ThermoFinnigan Italia) with a wavelength range of 200–300 nm. Data were acquired and analyzed with the ChromQuest program (ThermoQuest, Milan, Italy). Areas, retention times, and absorbance spectra of the peaks of sample chromatograms were compared with those of freshly prepared ultrapure standards, to determine the concentration of the various compounds at 267 nm (the upper limit of the MDA absorbance spectrum) and to identify different metabolites. Hemoglobin and the percentage of hemolysis were calculated with standard techniques [13] in a Jasco-685 double-beam spectrophotometer (Jasco Europe, Lecco, Italy). Briefly, Hb concentration was determined by adding 20 μl of whole blood to 5 ml of Drabkin reactive and measuring the absorbance at 540 nm. The percentage of hemolysis was determined adding 50 μl of heparinized whole blood to 5 ml NaCl 0.9% buffered at pH=7.4 and measuring the absorbance at 540 nm.

The blood energy-state levels were determined employing ATP, ADP, and AMP (detected by HPLC), and and the ECP was calculated according to the following formula: ECP=ATP+0,5 ADP/ΣNT, where ΣNT (=ATP+ADP+AMP) is the sum of the adenine nucleotide levels [14].

### Oxygen radical absorbance capacity assay

The oxygen radical absorbance capacity (ORAC) assay is based on the dose- and time-dependent decrease in the fluorescence intensity of β-phycoerythrin (β-PE) when oxidized by oxygen radicals [15]. It measures the antioxidant capacity of a substance—blood, vitreous, and aqueous humor in this case—in terms of its ability to inhibit or delay β-PE peroxidation.

Our assay was performed with the original method [16], with a few modifications [17]. AAPH [2,2’-Azobis(2-aminopropane)dihydrochloride] purchased from Polyscience (Warrington, PA) was used as the free radical generator, and β-PE was purchased from Sigma-Aldrich (St. Louis, MI). The final reaction mixture (2 ml) contained 1.750 ml of 75 μM phosphate buffer (pH 7.0) plus 0.100 ml of one of the following: 20 μM trolox (6-hydroxy-2,5,7,8-tetramethyl-2-carboxylic acid), which was used as the standard; a body fluid sample (blood, aqueous humor, or vitreous); or buffer alone (used as the reference). Beta-phycoerythrin (0.100 ml of a 34 mg/l solution) was placed in each well, and the oxidant reaction was started by adding 160 mM (0.050 ml per well) AAPH. Beta-PE fluorescence was measured with a Varian Cary Eclipse Fluorescence Spectrofotometer (Varian Australian PTY LTD, Victoria, Australia) at λ=546 nm (λ excitation) and λ=573 nm (λ is emission). Measurements were made every 2.5 min at 37 °C for 1 h or until the fluorescence variation dropped below 2%.

The ORAC of the sample was expressed as micromol trolox equivalents /g and calculated as ([As − Ab]/[At − Ab]) ka, where *As* is the area under the curve (AUC) of β-PE in the sample calculated with the Origin 2.8 integrating program (Microcal Software), *At* is the AUC of the trolox, *Ab* is the AUC of the control, *k* is the dilution factor (1:500 for the blood, 1: 100 for aqueous humor, 1:50 for vitreous), and *a* is the concentration of the trolox in mmol/l.

### Statistical analysis

Statistical analysis of differences between groups was performed with the ANOVA (ANOVA) test. Significance was set at p<0.05.

## Results

**Figure 1:**
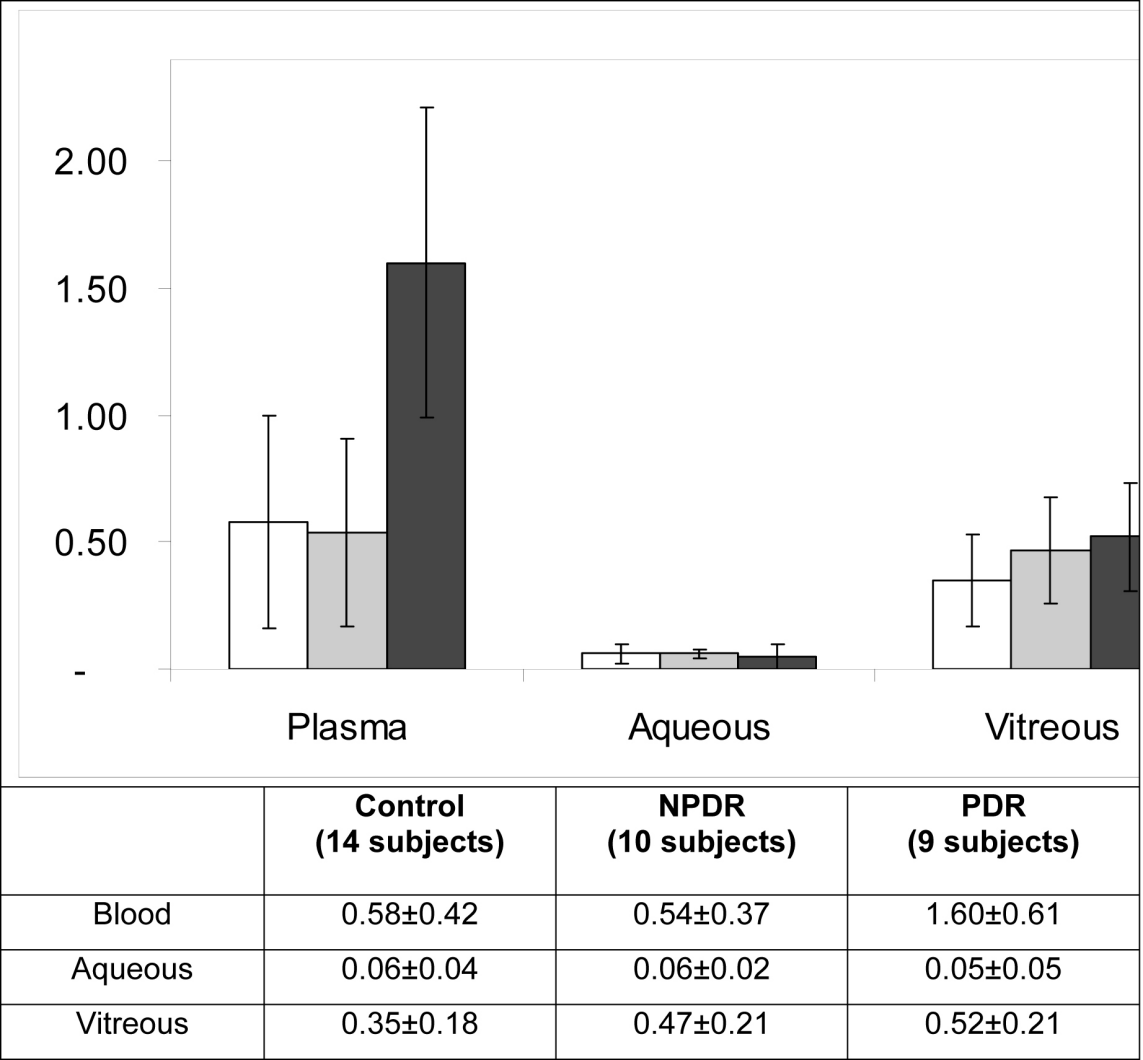
Malondialdehyde (MDA) levels in the blood, aqueous humor and vitreous of diabetic patients and controls. The levels of MDA were measured by High Performance Liquid chromatography in the blood, aqueous humor and vitreous of the control (white columns), non-proliferative diabetic retinopathy (NPDR; gray columns) and proliferative diabetic retinopathy (PDR; black columns) groups. Blood MDA levels in PDR patients were significantly increased over those of control and NPDR group (both, p<0.001). In the aqueous humor no significant differences between the groups were found. In the vitreous body, PDR patients, but not NPDR subjects, had increased MDA levels as compared with controls (p=0.050). Data are expressed in µmol/ml and represent mean±standard deviation (bars). ANOVA test was used.

As shown in Figure 1, blood MDA levels in diabetic patients were significantly increased over those of control patients (p<0.001), due to a highly significant increase of PDR values versus control group and versus NPDR values (both, p<0.001). At the level of the vitreous body, patients with PDR again had significantly increased MDA levels, compared to the controls (p=0.050), but there were no significant differences between the two groups of diabetic subjects and controls or between the NPDR subgroup of patients and controls. The analysis of MDA levels in the aqueous humor revealed no significant differences between any of the groups tested.

**Figure 2:**
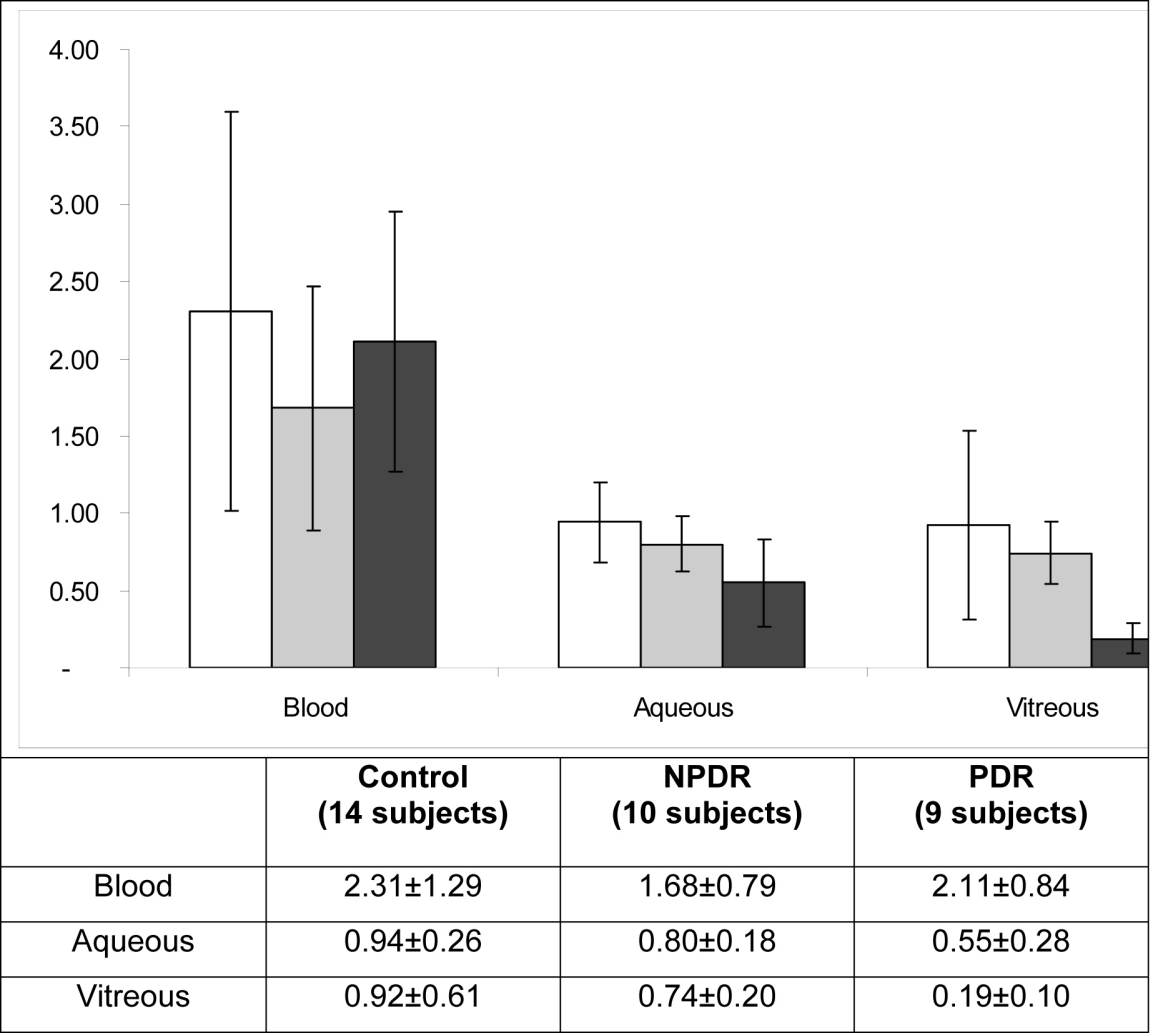
Total antioxidant capacity in the blood, aqueous humor, and vitreous of control and diabetic patients. The total antioxidant capacity (TAC) was measured by the oxygen radical absorbance capacity assay in the blood, aqueous humor and vitreous of controls (white columns) and of patients with non-proliferative diabetic retinopathy (NPDR; gray columns) and with proliferative diabetic retinopathy (PDR; black columns). The control group displayed significantly higher TAC levels than diabetic sub-groups in both the vitreous (p<0.001) and aqueous humor (p=0.003). No significant differences were observed in blood. Similar findings emerged from subgroup analyses. PDR patients had decreased TACs in the vitreous and aqueous humors as compared with control subjects (p=0.002 and p=0.003 respectively) and with the NPDR patient subgroup (p<0.001 and p=0.032 respectively). Data are expressed in µmol Trolox Equi/g and represent mean±standard deviation (bars). ANOVA test analysis was used.

On the whole, the control group also presented significantly higher TACs than diabetic subgroups in both the vitreous (p<0.001) and aqueous humors (p=0.003), but there were no significant differences in the TACs of blood samples from these groups (Figure 2). Similar findings emerged from subgroup analyses. PDR patients had decreased TACs in the vitreous and aqueous humors (but not in the plasma), compared to control subjects (p=0.002 and p=0.003, respectively) and with the NPDR patient subgroup (p<0.001 and p=0.032, respectively).

The ECP was measured only in the blood samples. On the whole, the control group (mean 0.85±0.04) exhibited significantly higher values than the NPDR and PDR groups, (ANOVA, p=0.002). In this case, the PDR and NPDR subgroups were not significantly different from one another, and both exhibited mean ECPs (NPDR patients, 0.80±0.05; PDR patients, 0.79±0.07) that were significantly lower than the control values (p=0.012 and p=0.016, respectively).

## Discussion

In this study, we attempted to characterize the oxidative stress and the total antioxidant capacities of blood and aqueous and vitreous humors in diabetic patients with or without proliferative retinopathy. Interestingly, in patients undergoing vitrectomy for PDR, levels of MDA in the blood and vitreous body (but not in the aqueous humor) were significantly higher than those found in nondiabetic controls and in diabetic patients with NPDR. Oxidants are highly reactive compounds with half-lives of a few seconds, and this seriously hinders their measurement in vivo [9]. However, many biomarkers have been developed to evaluate oxidative stress, including the breakdown products of peroxidized polyunsaturated fatty acids, such as MDA, which has proved to be both sensitive and reliable for this purpose [18]. Ion-pairing HPLC performed with tetrabutylammonium allows the simultaneous measurement of all nucleotides and corresponding deoxynucleosides with no chemical manipulation of samples other than perchloric acid deproteinization. This approach minimizes the risk that metabolite concentrations will be altered, and it provides clear, reliable, reproducible information on peroxidative damage and energy metabolism, both of which are important parameters in studies of ischemia and reperfusion injury [19]. Increased serum levels of MDA and conjugated dienes have been found in patients with diabetes [20], and in a recent study [9], serum MDA concentrations measured spectrophotometrically using a thiobarbituric acid-reacting substrate were found to be higher in patients with PDR than in those with NPDR or in healthy controls.

Our study is the first to use HPLC to explore this issue, and our findings confirm these previous reports on blood MDA, and extend this observation to the vitreous body. This suggests that high blood levels of MDA in diabetic patients reflect similar increases at the level of the vitreous body, and it points to the probable involvement of oxidative stress and lipid peroxidation in the progression of diabetic retinopathy to the proliferative form. In accordance with this hypothesis, we also found that patients with PDR had markedly reduced TACs at the levels of the vitreous (−80% versus controls) and aqueous (−40%) humors. Because it is relatively difficult to measure individual antioxidants separately, specific assays have been designed to measure the overall oxygen radical-scavenging capacity of fluid samples. The ORAC assay has been found to provide a good index of the total antioxidant capacity in patients with diabetes. Studies have shown that the plasma ORAC is reduced in diabetic patients in general, and patients with complications such as coronary artery disease and renal failure have lower values than those without complications [21]. However, reduced blood ORAC values are strongly associated with poor glycemic control in DM patients [21,22], and this may explain why the ORACs observed in our patients (with or without retinopathy) were not significantly different from those of the nondiabetic controls. Verdejo et al. also suggested that antioxidant defenses in the vitreous body were depressed in patients with PDR. Their conclusion was based on the results of assays of SOD activity and catalase levels [23], but the retina has several other mechanisms for minimizing oxidative stress, including those involving low molecular weight scavengers (i.e., α-tocopherol, glutathione, and ascorbic acid) and other enzymes like glutathione peroxidase [24]. In addition, every antioxidant system can exert its activity with different mechanisms and different efficiency, according to its chemical structure and the stage of the disease. For this reason, the TAC might be much more important than the concentration of single antioxidants. However, Izuta et al. [25] recently reported that the TAC of the vitreous is actually increased in patients with PDR. Their findings were not obtained with the ORAC assay we used with a PAO test, which provides quantitative estimates of the antioxidant capacity of a biologic fluid based on the reduction of Cu2^+^.

Interestingly, we also observed a reduced TAC in the aqueous humor of patients with PDR. Recent studies have demonstrated a close correlation between oxidative stress and morphological changes in the trabecular meshwork [26-28], suggesting that anterior chamber involvement in patients with PDR may be caused partly by redox-state imbalances.

Finally, the blood of our diabetic patients exhibited significantly reduced energy charge potentials, compared to controls, and interestingly enough, this difference was not limited to the patients with proliferative retinopathy. The ECP represents the balance between energy-producing and energy-consuming reactions, and decreased values generally reflect insufficient production of ATP via the oxidative and phosphorylative activities of the mitochondria. Our finding indicates that in diabetic patients, the blood energy supply is deficient even before the onset of retinopathy, and its reduction may be a primary event in the development of the complications associated with the disease.

On the whole, these data indicate that alteration of the redox state and the energy potential contribute to the development of diabetic retinopathy, although the mechanisms by which oxidative stress triggers this event have not been fully elucidated. Oxidative stress not only creates a vicious cycle of damage to membrane lipids, proteins, and DNA, it also amplifies ROS production by also activating other metabolic pathways that are involved in the development of diabetic retinopathy [8]. These include the polyol pathway [29], the protein kinase C pathway [30,31], the hexosamine biosynthesis pathway [32], and the advanced glycation end product pathway [33], the last of which has been implicated in basement-membrane thickening [34] and the loss of pericytes and endothelial cells that it causes. ROS are also known to promote the breakdown of the blood-retinal barrier and to alter retinal blood flow by modulating production of vasoactive effector molecules such as endothelin-1 [35]. In addition, oxidative stress also upregulates the retinal expression of VEGF [36] and mediates the hyperglycemia-induced effects of VEGF that give rise to the microvascular complications of diabetes [37]. Recently, Izuta [25] found a positive correlation between N-hexanoyl-lysine (a lipid peroxide) and VEGF concentrations in the vitreous bodies of patients with PDR, thus confirming that oxidative stress induced by increased ROS generation may play a pivotal role in the upregulation of VEGF in the vitreous body in PDR patients.

The main limitation of our study is the small number of patients examined, which reflects difficulties in recruiting patients with the clinical characteristics used as selection criteria.

Taken together, our results strongly suggest that the development of diabetic retinopathy is associated with high-level oxidative stress and diminished antioxidant defenses.
